# Mechanism of the Zn(II)Phthalocyanines’ Photochemical Reactions Depending on the Number of Substituents and Geometry

**DOI:** 10.3390/molecules21050635

**Published:** 2016-05-14

**Authors:** Leandro Henrique Zucolotto Cocca, Mehmet Menaf Ayhan, Ayşe Gül Gürek, Vefa Ahsen, Yann Bretonnière, Jonathas de Paula Siqueira, Fernando Gotardo, Cleber Renato Mendonça, Catherine Hirel, Leonardo De Boni

**Affiliations:** 1 Instituto de Física de São Carlos, Universidade de São Paulo, CP 369, 13560-970 São Carlos-SP, Brazil; leandro.cocca@usp.br (L.H.Z.C.); jonathasusp@gmail.com (J.P.S.); gotardo.fernando@gmail.com (F.G.); crmendon@ifsc.usp.br (C.R.M.); 2 Department of Chemistry, Faculty of Science, Gebze Technical University, P. O. Box 141, Gebze, 41400 Kocaeli, Turkey; menafayhan@gtu.edu.tr (M.M.A.); gurek@gtu.edu.tr (A.G.G.); ahsen@gtu.edu.tr (V.A.); 3 Laboratoire de Chimie de l’ENS de Lyon, CNRS UMR 5182, Université Lyon I, ENS de Lyon, 46 allée d’Italie, 69364 Lyon cedex 07, France; yann.bretonniere@ens-lyon.fr

**Keywords:** Zn(II)phthalocyanine, optical nonlinearities, luminescence

## Abstract

In this work, the synthesis and the nonlinear absorption and population dynamics investigation of a series of zinc phthalocyanines (ZnPcs) dissolved in chloroform are reported. In order to determine the relevant spectroscopic parameters, such as absorption cross-sections of singlet and triplet excited states, fluorescence relaxation times, intersystem crossing, radiative decay and internal conversion, different optical and spectroscopic techniques were used. By single pulse and pulse train Z-scan techniques, respectively, singlet and triplet excited states‘ absorption cross-section were determined at 532 nm. Furthermore, the intersystem crossing time was obtained by using both techniques combined with the fluorescence lifetime determined by time-resolved fluorescence. The radiative and internal conversion rates were determined from the fluorescence quantum yield of the samples. Such spectroscopy parameters are fundamental for selecting photosensitizers used in photodynamic therapy, as well as for many other applications.

## 1. Introduction

Phthalocyanines are organic molecules and remarkable molecular materials [1,2,3] whose structures and properties are similar to porphyrins [4]. Especially, in symmetric and asymmetric substituted phthalocyanines [5,6,7], the ability to tailor their properties by varying the metal center or by modifying the peripheral substituent allows large flexibility [8] from the applications point of view, such as in photodynamic therapy [9,10,11,12] and organic solar cells [13,14,15].

**Figure 1:**
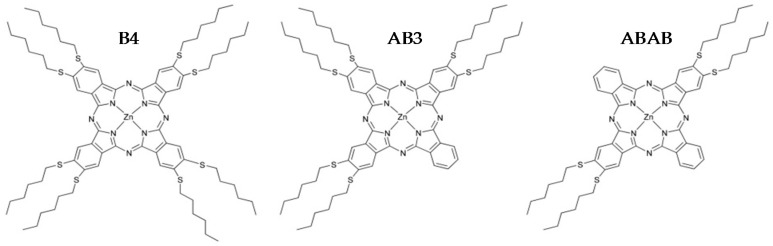
Molecular structure of **B4**, **AB3** and **ABAB** ZnPc, respectively.

In this context, the fundamental understanding of their unique optical properties, such as luminescence and optical nonlinearities, is a cornerstone; this article lies on this perspective. Zinc phthalocyanines [16,17] present interesting fluorescence properties, high triplet yield, long lifetime and optical nonlinearities [18,19,20,21], such as large nonlinear absorption due to the intense reverse saturable absorption [22] (RSA), which led us to design 2,3,9,10,16,17,23,24-octakis(hexylthio)phthalocyaninato)zinc (**B4**), (2,3,9,10,16,17 hexakis(hexylthio)phthalocyaninato)zinc (**AB3**) and (2,3,16,17-tetrakis(hexylthio)phthalocyaninato)zinc (**ABAB**) varying the number of thioalkyl substituents to study the influence of the molecule geometry and electronic structure (see Figure 1).

Herein, the synthesis and characterization of **B4**, **AB3** and **ABAB** are reported, as well as their photophysical and nonlinear optical properties. For the analysis of optical nonlinearities dynamics and excited states’ properties, single pulse [23] and pulse trains [24] Z-scan techniques were used, both at 532 nm. Additionally, a white light continuum Z-scan [23,24,25,26,27] was employed to measure the excited state absorption spectrum in the visible region (450 nm up to 750 nm). Absorption spectra of all of the target zinc (II) phthalocyanines (ZnPcs) present saturable absorption (SA) and reverse saturable absorption; both depend on the excitation wavelength. In order to obtain RSA efficiency, different spectroscopic techniques were combined to analyze the relaxation time, intersystem crossing time, ground and excited state cross-sections, because the efficiency of the RSA depends on these parameters.

## 2. Results and Discussion

### 2.1. Synthesis

**ABAB** and **AB3** were obtained in a 30% and a 24% yield, respectively, by direct metal insertion into **ABAB-H2** and **AB3-H2** by refluxing anhydrous zinc acetate in hexanol, in the presence of a strong base 1,8-diazabicyclo-[5,4,0]undec-7-ene (DBU); whereas metalation and phthalocyanine formation was achieved directly for **B4**, by tetracyclomerization of 4,5-hexylthiophthalonitrile. The position of the absorption peaks in the infrared (IR), UV-VIS and ^1^H-NMR data are in agreement with literature for **B4 and** with the proposed structures **for AB3** and **ABAB** (see Supplementary Materials Figuers S5–S7 and S12–S14).

### 2.2. Ground State Electronic Absorption and Steady-State Fluorescence Measurements

**Figure 2:**
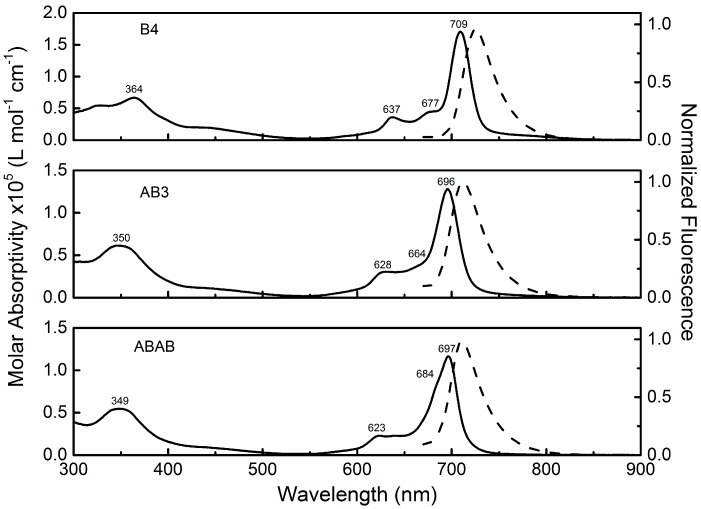
Molar absorption (**solid lines**) and normalized fluorescence (**dashed lines**) spectra of **B4**, **AB3** and **ABAB** dissolved in chloroform.

The molar absorptivity (solid lines) and fluorescence (dashed lines) spectra are presented in Figure 2. The sharp and single Q-bands confirm that phthalocyanines dissolved in chloroform are not aggregated at the used concentration. The absorption spectrum for **B4** is similar to the one reported in [17], in which the solvent was THF, with a polarity index similar to chloroform. In the case of the low symmetrical **AB3** and **ABAB**, the Q_00_ band does not present a significant split (shoulder at 684 for ABAB). As reported by Nemykin and co-workers [28], this suggests that the two LUMOs (LUMO and LUMO + 1) in these compounds are accidentally nearly degenerate. In the case of D_2h_, a large split is expected [28]. Furthermore, a small red shift of the Q-band is observed for **B4** at approximately 700 nm (of about 5 nm) nm when compared to **AB3** and **ABAB**, indicating a decrease in the HOMO-LUMO energy. This happens by the increase of the electron donation to the main ring due to the sulfur atom attached to the hexyl chains. In the case of **B4**, one can observe considerable changes in the shape of the spectrum, for both Soret (~350 nm) and Q-bands (630 nm, 675 nm and 705 nm) when compared to the other two phthalocyanines. The bands are more defined, and this occurs by the increase in the molecular symmetry. Additionally, the reduction of the strong electron-donor group number, from eight (**B4**) to six (**AB3**) and, finally, four (**ABAB**), directly affects the oscillator strength of all electronic transitions. One can observe, for example, a decrease in the molar absorptivity (ε) from 1.7 to 1.3 and 1.2 (×10^5^ L mol^−1^·cm^−1^), respectively from **B4** to **AB3** and, finally, to **ABAB**, as the number of hexylsulfonyl groups decreases. From these spectra, recorded at a well-known molecular concentration, the ground state absorption cross-section σ_01_(λ) cm^2^ was obtained [29]. This quantity will be needed to describe the population dynamics observed in ZnPcs excited states employing a rate equation model. The fluorescence spectra displays a single emission band that ends at approximately 800 nm, a behavior similar to the ones already reported in the literature [16,17].

### 2.3. Fluorescence Quantum Yield Measurements

The values of the fluorescence quantum yields determined for all three samples dissolved in chloroform are: $\phi_{f}=9.6\%$ for **B4**, $\phi_{f}=18.1\%$ for **AB3** and $\phi_{f}=20.7\%$ for **ABAB**, which show an increase of the fluorescence quantum yield proportionally to the decrease of the thiohexyl chains. Such behavior can be explained by the changes in charge symmetry from **B4** to **ABAB** and the decrease of the number of electron donor groups attached to the main ring. By reducing the hexylsulfonyl groups, the charge density on the ring decreases, increasing the radiative decay rates and the fluorescence quantum yield [4]. Topal and coworkers [17] observed a similar behavior when they compared **B4** with an almost identical phthalocyanine; the only difference was the replacement of the sulfur atoms by oxygen ones. Due to the lower electron-donating strength caused by the replacement of the sulfur, the fluorescence quantum yield was significantly increased from 13% to 19%.

### 2.4. Fluorescence Lifetimes (Time-Resolved Measurements)

Within our experimental resolution, a single exponential decay was obtained for all samples dissolved in chloroform, with a lifetime of about 3.1 ± 0.3 ns. Such lifetimes are in agreement with the ones reported for ZnPcs dissolved in chloroform [16] and are close to the one reported for **B4** dissolved in THF [17]. Once the fluorescence lifetime and quantum yields are determined, one could estimate the radiative rate $k_{r}=\phi_{f}/\tau_{f}$ from all samples dissolved in chloroform. This rate constant will be later presented in Table 1.

### 2.5. Excited State Absorption Cross-Section: Z-Scan

In order to determine the excited state absorption cross-section (singlet and triplet ones) and the relaxation rates’ processes, such as intersystem crossing time, of all ZnPcs molecules dissolved in chloroform, we used three different Z-scan [23] setups; the conventional single pulse Z-scan technique, the pulse train Z-scan technique (PTZS), both at 532 nm, and the white light continuum Z-scan (WLCZS). The first two techniques are used to map the population dynamics between singlet and triplet states, while WLCZS is used to determine the singlet excited state in a larger wavelength range, from 450 up to 800 nm. It is important to mention that the changes in the normalized transmittance obtained with the Z-scan are a mirror of how the absorption of the samples changes over time when high intensity laser pulses are employed as excitation.

**Figure 3:**
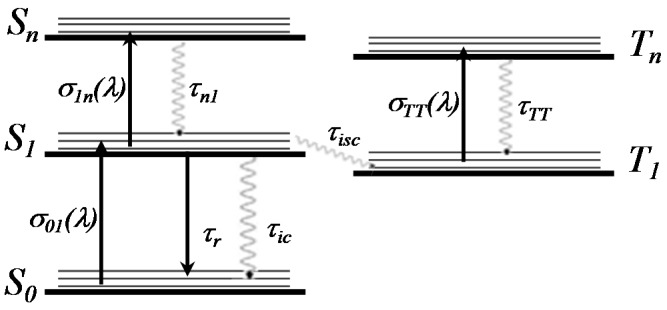
Jablonski five-level energy diagram used to model the excited state absorption of ZnPcs’s.

Due to the high intensity used, a significant electronic population can be transferred from the ground singlet state to excited singlet ones and, if it is allowed, to triplet excited states, as well. This excited population changes the transmittance of the laser pulse through the sample according to the excited state absorption cross-sections. Therefore, in order to describe the changes in the normalized transmittance, obtained by the three different Z-scan methods, a five-level energy diagram (Jablonski) was employed, as illustrated in Figure 3.

Once the absorption cross-sections (σ*’s*) of the states are wavelength dependent and intrinsic to each molecule, it is only necessary to solve the population dynamics over the five energy levels as a function of the pulse fluence. Thus, one should simulate a dynamical absorption coefficient, α(t,λ), which depends only on the fraction of the population in each state ($n_{i}\left(t\right)$) and then determine the excited state absorption cross-section by using the following equation:

(1) $$\alpha \left(t,\lambda \right)=N\left[n_{S0}\left(t\right)\sigma_{01}\left(\lambda \right)+n_{S1}\left(t\right)\sigma_{1n}\left(\lambda \right)+n_{T1}\left(t\right)\sigma_{TT}\left(\lambda \right)\right]$$

in which *N* is the number of molecules per cm^3^.

**Figure 4:**
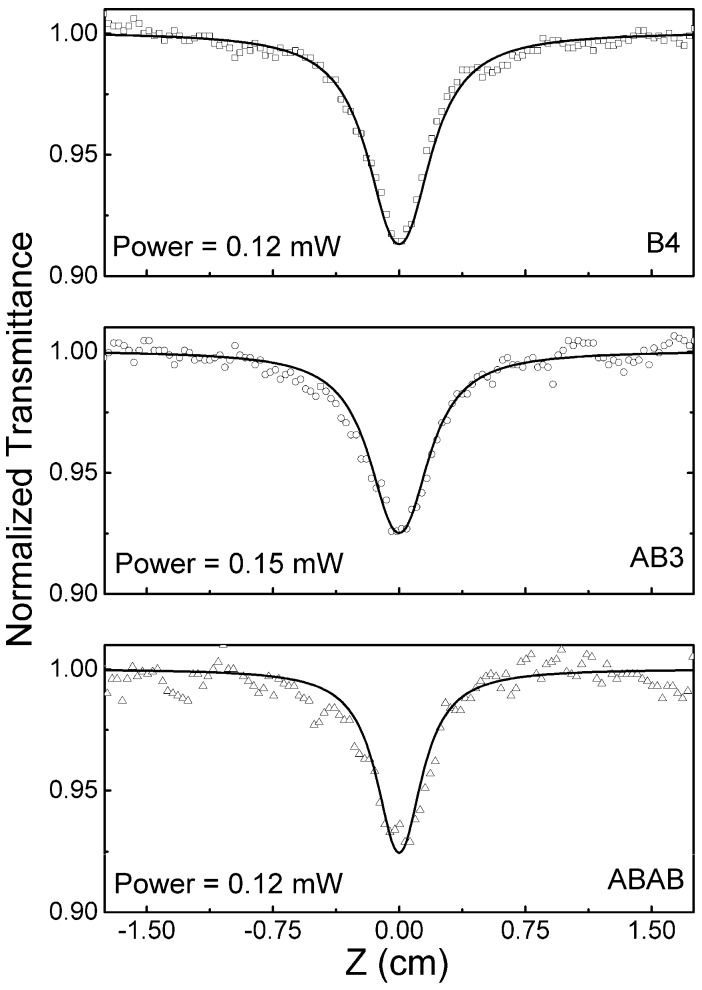
Normalized transmittance obtained by the single pulse Z-scan at 532 nm (symbols). The solid line represents the theoretical fitting obtained by using the rate equations (Equation (2)) and Equation (1).

In Equation (1), it is possible to see that the absorption coefficient depends on the singlet and triplet states. The only known parameter of the system depicted in Figure 3, until now and obtained through linear spectroscopy, is the ground state absorption cross-section, $\sigma_{01}\left(\lambda \right)$, which was determined through the Beer-Lambert law and corresponds to a value at 532 nm of about $1.12\;\times \;10^{-17}\;{\text{cm}}^{2}$ for **B4**, $0.75\;\times \;10^{-17}{\text{ cm}}^{2}$ for **AB3** and $0.48\;\times \;10^{-17}{\text{ cm}}^{2}$ for **ABAB**. Therefore, in order to determine each state separately and to reduce the number of adjusted parameters, we initially simulated the experimental results obtained with single pulse and WLC Z-scans. For both cases, we used only the three singlet energy levels shown in Figure 3 ($S_{0}$, $S_{1}$ and $S_{n}$). In this case, the triplet states were neglected because the single and WLC pulses have durations of about 100 ps and 4 ps, respectively. During these times, which are much shorter than the intersystem crossing time (in the order of ns for ZnPc [16]), the population fraction transferred for the triplet ($n_{T1}\left(t\right)$) is negligible, simplifying Equation (1) for only singlet terms. Thus, the rate equations necessary to describe the population dynamics in the three singlet energy levels are:

$$\frac{dn_{s0}\left(t\right)}{dt}\;=-\;W_{01}n_{S0}\left(t\right)+\;k_{f}n_{S1}\left(t\right)$$

$$\frac{dn_{s1}\left(t\right)}{dt}=W_{01}n_{S0}\left(t\right)-W_{1n}n_{S1}\left(t\right)-k_{f}\;n_{S1}\left(t\right)+k_{n1}n_{Sn}\left(t\right)$$

(2) $$\frac{dn_{Sn}\left(t\right)}{dt}=W_{1n}n_{S1}\left(t\right)-k_{n1}n_{Sn}\left(t\right)$$

in which $n_{s0}\left(t\right)$, $n_{s1}\left(t\right)$ and$\;n_{s2}\left(t\right)$ are the fraction of the population in the ground state, first and second singlet excited states, respectively. k=1/τ are the decay or relaxation rates; $W_{ii\prime }=I\left(t\right)\sigma_{ii\prime }/h\nu$ is the transition rate between energy levels of the singlet states; I(t) is the intensity in W/cm^2^; h is the Plank constant; and ν is the light frequency in Hz. The fluorescence rate ($k_{f}=1/\tau_{f}$) was determine by time-resolved measurements, already described. The relaxation time of the second excited state to the first excited state is known to be around 300 fs for phthalocyanines and porphyrins [30,31]. Solving numerically the rate equations and using only as an adjustable parameter the excited state absorption cross-section, $\sigma_{1n}\left(\lambda \right)$, we were able to model (solid lines) the single-pulse Z-scan signatures at 532 nm (symbols), as shown in Figure 4. The experimental results measured at 532 nm with a 100-ps pulse laser show a decrease in the normalized transmittance (NT) at the focal position (Z = 0 cm). A decrease in the NT only occurs if the excited state has an absorption cross-section higher than the one of the ground state. A similar behavior of the decrease of the NT at 532 nm was also reported for symmetric ZnPc with only hydrogen as the peripheral groups [16].

The value obtained throughout the fittings for the singlet excited state absorption cross-sections were $\sigma_{1n}=3.1\pm 0.4\;\times \;10^{-17}\;{\text{cm}}^{2}$ for **B4** (2.8-times higher than the ground state absorption cross-section), ${\text{σ}}_{1n}=2.4\pm 0.2\;\times \;10^{-17}\;{\text{cm}}^{2}\;$for **AB3** (3.2-times higher than the ground state absorption cross-section) and $\sigma_{1n}=1.9\pm 0.4\;\times \;10^{-17}\;{\text{cm}}^{2}$ for **ABAB** (four-times higher than the ground state absorption cross-section). Consequently, they are good candidates as optical limiters on the picoseconds regimes at 532 nm, especially the **ABAB** crosswise molecule that presents a four-fold singlet excited state absorption cross-section compared to the ground state. This can be explained by the decrease in the red shift of the ground state absorption due to the reduction of the donor groups. At the 530-nm region, the ground state absorption has the lowest value for **ABAB**, increasing the ratio between the excited and ground absorption cross-sections.

**Figure 5:**
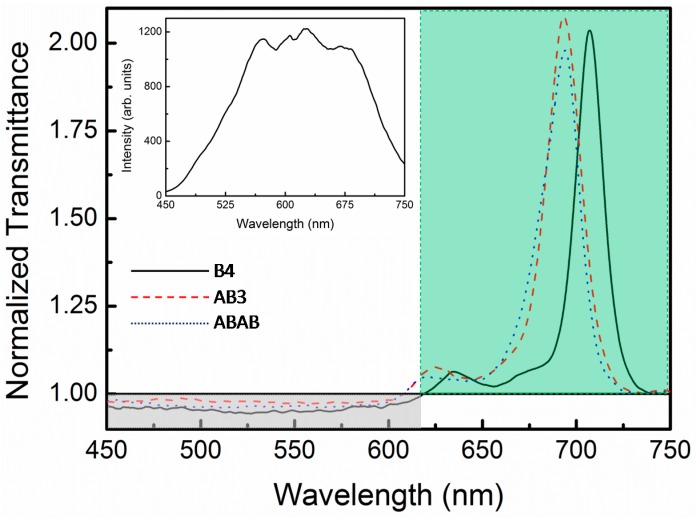
Normalized transmittance spectra obtained by using the white light continuum Z-scan. Each one of the three curves represents the nonlinear transmittance obtained for **B4**, **AB3** and **ABAB** dissolved in chloroform. The inset shows the white light continuum spectrum used in the WLCZS technique.

Similar experimental signatures were obtained by using the WLCZS technique. However, this technique provides a wider spectral view with a high resolution of the excited state absorption spectrum because of the continuous broadband of the WLC (see the inset of Figure 5). This can be observed in Figure 5 by the NT spectra of **B4**, **AB3** and **ABAB** dissolved in chloroform. In Figure 5, changes in samples’ transmittance as a function of the wavelength, at the focal position of the WLCZS, can be appreciated. It is possible to identify three different main regions; one between 620 and 750 nm (marked by a green area), which corresponds to the same region of the Q-band (see Figure 2, linear absorption), presents an increase in the nonlinear optical transmittance. This phenomenon is explained by the excited state absorption cross-section magnitude, $\sigma_{1n}$. The nonlinear optical transmittance increases only if $\sigma_{1n}$ presents a value lower than the ground state one $\sigma_{01}$, meaning that the excited state is more transparent to this spectral region; such an effect is known as saturable absorption. In the opposite way, in the region between 450 and 600 nm (marked by a gray area), the nonlinear transmittance decreases to a value close to 0.94. The decrease in the transmittance is explained only if $\sigma_{1n}$ is higher than the ground state cross-section, *i.e.*, the excited state is less transparent in this spectral region; this is the reverse saturable absorption effect and is the principle for optical limiting. In the last region, around 615 nm, the nonlinear transmittance is close to one and corresponds to the region in which $\sigma_{1n}$ and $\sigma_{01}$ present the same values, meaning that the population transferred to the excited state from the ground state does not change the absorption of the sample.

Using the normalized transmittance spectra obtained through WLCZS, we were able to calculate the excited state absorption cross-section spectra for all three samples by using Equation (1) and the same rate equation model describe in Equation (2), which also simulates the population fraction as a function of the time for each wavelength, during the interaction between the sample with the white light continuum beam of the WLCZS experiment.

**Figure 6:**
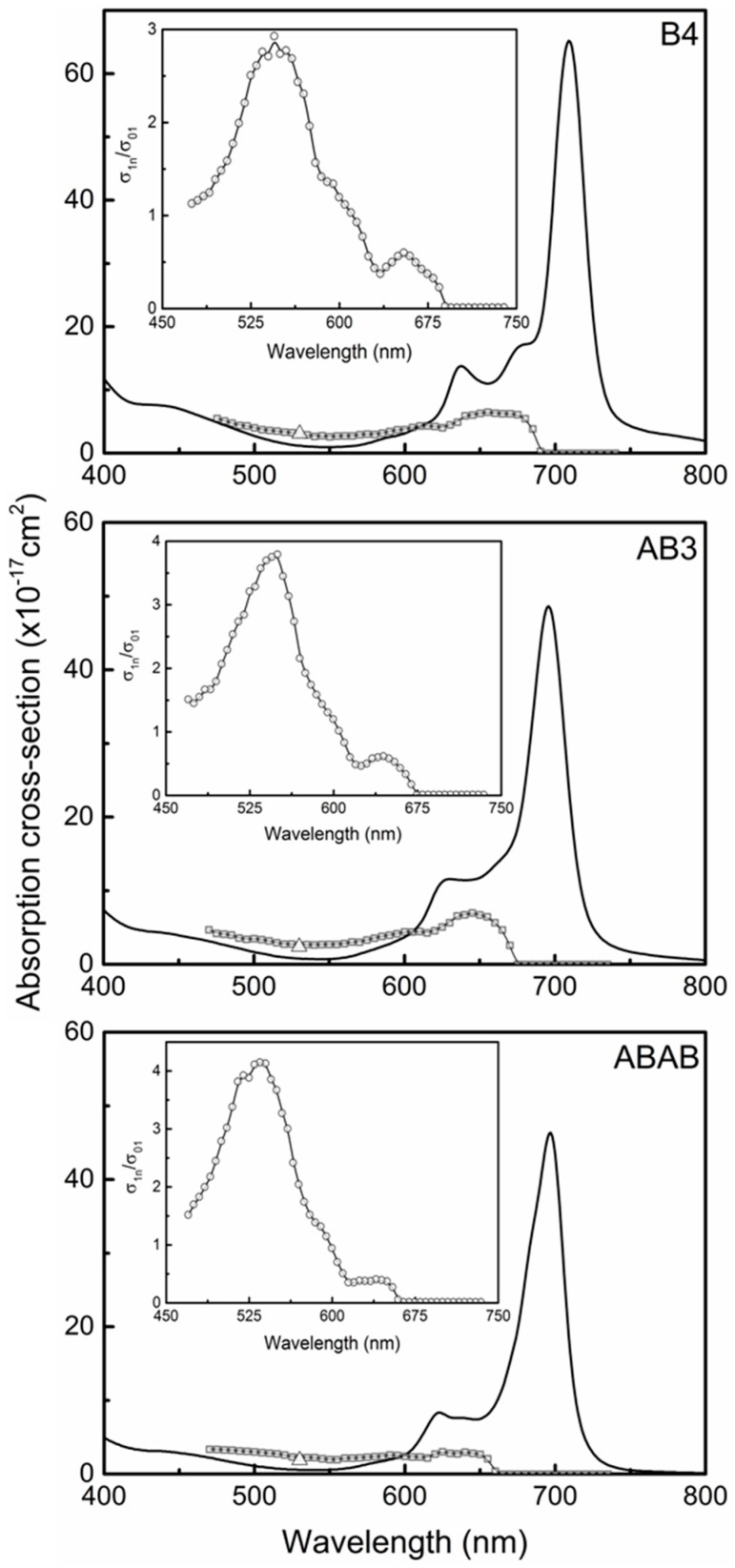
Ground (solid line) and excited state (open square) absorption cross-section spectra for **B4**, **AB3** and **ABAB**. The insets show the ratio (open circles) between the excited and ground state absorption cross-section. The open triangle represents the excited state absorption cross-section at 532 nm, obtained with the single-pulse Z-scan technique.

After adjusting the NT depicted in Figure 5, one obtains the excited state (open squares) absorption cross-section spectrum for each ZnPc sample displayed in Figure 6. $\sigma_{1n}\left(\lambda \right)$ was plotted together with the ground state absorption cross-section (solid line) to make clear the regions in which the samples became more or less transparent at the excited state. The spectral resolution of $\sigma_{1n}\left(\lambda \right)$ is 5 nm. The excited state absorption cross-section at 532 nm, obtained with single (open triangle) and WLC (open squares) Z-scan techniques, are in good agreement.

In Figure 6, the three spectra (open squares) present an excited state absorption cross-section close to zero for wavelengths longer than 680 nm up to 750 nm, which indicates that at this range, there is no observable transition from the first excited state. However, for wavelengths shorter that 680 nm until 450 nm, $\sigma_{1n}\left(\lambda \right)$ exhibits nonzero values, indicating that the transition $S_{1}\to S_{n}$ is taking place at this region. It is interesting to notice that the small red shift observed in the linear absorption is also present for sample **B4** when compared to **AB3** and **ABAB** dissolved in chloroform, as clearly observed at the wavelength in which the excited state absorption cross-section is different than zero. In the case of **B4**, it happens at 690 nm, for **AB3** at 670 nm and for **ABAB** at 655 nm. Moreover, for the insets of Figure 6, which depict the ratio $\sigma_{1n}\left(\lambda \right)/\sigma_{01}\left(\lambda \right)$, a maximum ratio value is observed at approximately 540 nm. The shapes of $\sigma_{1n}\left(\lambda \right)/\sigma_{01}\left(\lambda \right)$ can be considered very close to each other, which indicates that the number of hexyl chains linked to the macrocycle by a sulfur atom does not significantly change the excited state electronic transition.

The next step of our photophysical analyze was to obtain the intersystem crossing (ISC) time from the $S_{1}\to T_{1}$ system and triplet state absorption cross-section $\sigma_{TT}$, which mediates the transition $T_{1}\to T_{n}$ for **B4**, **AB3** and **ABAB** dissolved in chloroform. To observe the mechanism of ISC the pulse train Z-scan (PTZS) was used, which allows monitoring population cumulative effects. In other words, this technique pumps and probes the populations dynamics over the states involved just by measuring changes in the transmittance of the sample as a function of pulses of the train.

**Figure 7:**
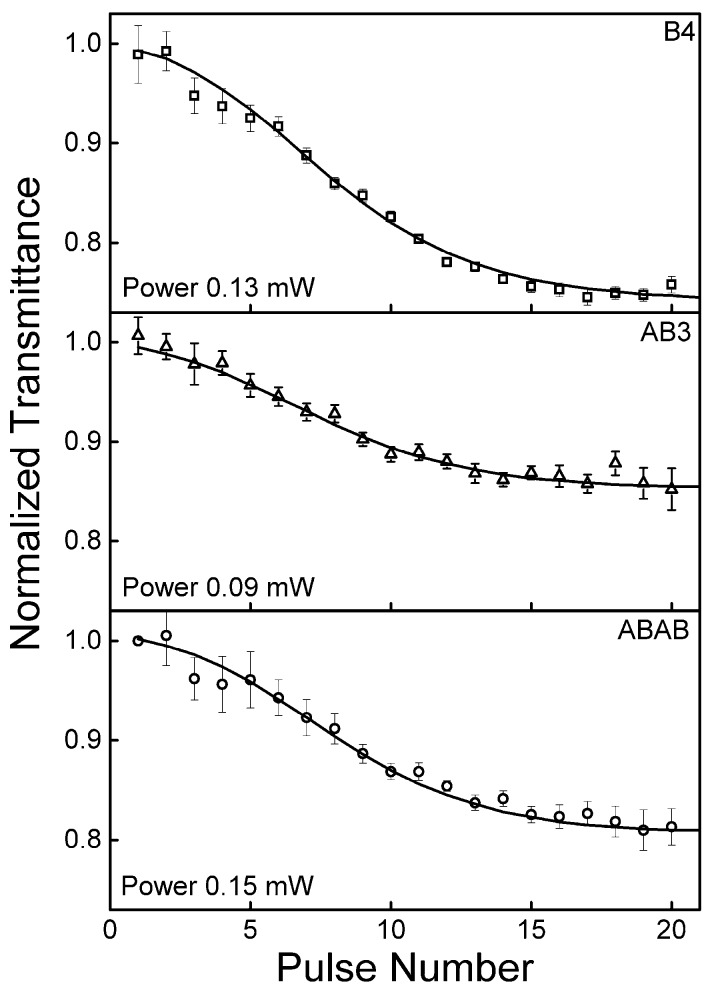
Experimental normalized transmittance (symbols) along the pulse train for **B4**, **AB3** and **ABAB** dissolved in chloroform measured at 532 nm. The solid lines represent the best fits obtained with the rate equation described by Equation (3).

As the fluorescence lifetime and the absorption cross-sections (σ’*s*) of the singlet states at 532 nm are known for each molecule, the population dynamics over the five energy levels can be solved as a function of the pulse fluency given by the pulse train pattern. The dynamical absorption coefficient, α(t,532 nm), presented in Equation (1), depends only on the fraction of the population in each state ($n_{i}\left(t\right)$) that can be determined using the following rate, according to the five energy level diagram:

$$\frac{dn_{S0}\left(t\right)}{dt}\;=\;-W_{01}n_{S0}\left(t\right)+\left(k_{f}-k_{isc}\right)n_{S1}\left(t\right)$$

$$\frac{dn_{S1}\left(t\right)}{dt}=W_{01}n_{S0}\left(t\right)-W_{1n}n_{S1}\left(t\right)-k_{f}\;n_{S1}\left(t\right)+k_{n1}n_{Sn}\left(t\right)$$

(3) $$\frac{dn_{Sn}\left(t\right)}{dt}=W_{1n}n_{S1}\left(t\right)-k_{n1}n_{Sn}\left(t\right)$$

$$\frac{dn_{T1}\left(t\right)}{dt}=\;k_{isc}n_{S1}\left(t\right)+\;k_{TT}n_{Tn}\left(t\right)-W_{TT}n_{T1}\left(t\right)$$

$$\frac{dn_{TT}\left(t\right)}{dt}=W_{TT}n_{T1}\left(t\right)-\;k_{TT}n_{Tn}\left(t\right)$$

in which $n_{S0}\left(0\right)$ = 1, $n_{Si}$ are the fraction population on the singlet states, $n_{Ti}$ are the fraction population on the triplet states, $W_{ii\prime }=I\left(t\right)\sigma_{ii\prime }/h\nu$ are the transition rate between energy levels of the singlet and triplet states. $k_{i´i}\;$represents the decay or relaxation rates and is defined as $\tau_{i´i}=\;1/k_{i´i}\;;\;k_{f}$ is the decay rate of fluorescence (=$1/\tau_{f}$); and $k_{isc}$ is the rate of intersystem crossing ($=1/\tau_{isc}$). Additionally, we used the condition $\overset{\infty }{\underset{i=1}{\sum }}n_{i}\left(t\right)=1$ to solve the set of rate equations. The first three terms in Equation (3) set are similar to the ones in Equation (2). Considering now that the terms related to the triplet states cannot be neglected in the pulse train time interaction with the sample, two new terms are added to describe the population dynamics on the triplet states. Solving numerically this set of rate equations and using as only adjustable parameters the triplet excited state absorption cross-section, $\sigma_{TT}\left(532\;nm\right)$, and the intersystem crossing time, $\tau_{isc}$, we were able to model (solid lines) the PTZS experimental results at 532 nm (symbols), as depicted in Figure 7. One can observe a decrease of the NT as a function of the pulse number. This effect is caused by population transferred from the first excited state to the triplet state and describes that $\sigma_{TT}\left(532\;nm\right)$ is higher than the ground state one. In other words, molecules in the triplet state absorb more light than the ones on the ground state at 532 nm.

In order to understand how this process takes place, triplet state absorption cross-section and intersystem crossing time can be determined. The interaction between the pulse train and the samples is explained as follows; after the first interaction between the molecule and the first pulse train of pulses, excitation occurs from $S_{0}$ to $S_{1}$, transferring the population to $S_{1}$. Thus, in $S_{1}$ molecules can relax back to the ground state $S_{0}$ or undergo the triplet state $T_{1}$. When the next pulse (13 ns after the first pulse) of the train excites the sample, there are populations already in the other states, including a small fraction in the triplet state. This mechanism continues exciting the samples as the whole pulse train passes through the sample. As a consequence, during the interaction between the sample and each individual pulse of the train, the population is transferred to the triplet state where it is accumulated due to the long lifetime of the $T_{1}$ state. Based on this, the fractions of molecules in each individual state will absorb light with different probabilities, given by the σ of the respective states. In this way, the transmittance of the sample changes as the fraction of population changes over time, giving a cumulative effect on the NT as a function of the time, as observed in Figure 7.

Since the rate equation model takes into account the pulse train profile as the input fluence and the spectroscopic parameters already determined, the only way to obtain the proper fit (solid lines) is by considering the ISC time and the triplet excited state absorption cross-section as the only adjustable parameter in the model. As a result, the best fits provided intersystem crossing times: $\tau_{isc}=16.5\;\pm 0.5\;\text{ns}$ for **B4**, $\tau_{isc}=15.1\;\pm 0.6\;\text{ns}$ for **AB3** and $\tau_{isc}=9.4\;\pm 0.5\;\text{ns}$ for **ABAB**. Additionally, the values of the absorption cross-section of the triplet state are: $\sigma_{TT\;}=3.4\pm 0.4\;\times \;10^{-17}{\text{ cm}}^{2}\;$(three-times higher than the ground state cross-section) to **B4**, $\sigma_{TT\;}=2.7\pm 0.3\;\times \;10^{-17}{\text{ cm}}^{2}$ (3.6-times higher than the ground state cross-section) to **AB3** and $\sigma_{TT\;}=2.2\pm 0.1\;\times \;10^{-17}{\text{ cm}}^{2}$ (4.6-times higher than the ground state cross-section) to **ABAB**. The values obtained for the ZnPcs studied in this work are in agreement to the ones reported for symmetric ZnPc [16].

At the end, triplet formation quantum yield is calculated by $\phi_{T}=\tau_{f}/\tau_{isc}$, providing: $\phi_{T}=19\%$ to **B4**, $\phi_{T}=21\%$ to **AB3** and $\phi_{T}=32.0\%$ to **ABAB**. Using $\phi_{T}$ and $\phi_{f}$, the nonradiative quantum (internal conversion) yield were obtained by using $\phi_{nr}=1-\left(\phi_{T}+\phi_{f}\right)$, giving $\phi_{nr}=\;71.7\%\;$to **B4**, $\phi_{nr}=60.8\%$ to **AB3** and $\phi_{nr}=47.4\%\;$to **ABAB**, all dissolved in chloroform.

The radiative and nonradiative decay times are calculated, respectively, using $\tau_{r}=\tau_{f}/\phi_{f}$ and $\frac{1}{\tau_{nr}}=\frac{1}{\tau_{f}}-\frac{1}{\tau_{r}}-\frac{1}{\tau_{isc}}$. As result, the radiative decays ($\tau_{r}$) are 32.3 ns for **B4**, 17.7 ns for **AB3** and 14.5 ns for **ABAB** all dissolved in chloroform. The nonradiative decay times are 4.4 ns for **B4**, 5.3 ns for AB3 and 6.5 ns for **ABAB**. In Table 1, we summarized the parameters determined in this work.

Looking at Table 1, one can observe that the intersystem crossing time decreases as the number of hexyl chains linked to the macrocycle by a sulfur atom decreases, increasing the probability of molecules undergoing intersystem crossing to a triplet state, as can be visualized in the triplet formation quantum yield $\phi_{T}$. An important information is that the main relaxation pathway for the first singlet excited state happens via internal conversion, and the highest quantum yield of this process was obtained for **B4**, indicating that the number of hexyl chains is vital to control it. This behavior can be explained by the so-called “loose bolt” effect [32,33], which should appear in the σ C-H bonds of the hexyl chains. The stretching vibration is similar to a “loose bolt” in some moving parts of a machine, which tends to be set in motion by other moving parts. According to this effect, the “loose bolt” mechanism accelerates internal conversion once electronic energy is lost through C-H vibrations. In this way, this effect is expected to be less pronounced in **ABAB** due to the reduced number of hexyl chains, as was observed in Table 1.

Phthalocyanines bearing indium, gallium, thallium, lead and gadolinium have also been studied on the optical limiting point of view [34,35,36,37]. The nonlinear optical effects (excited state absorption cross-section) presented here are on the same order of magnitude of those reported in the literature [34,35,36,37]. For example, Yuksek and coworkers [34] studied indium and gallium phthalocyanines in solution and in a co-polymer host using 4-ns laser pulses at 532 nm to obtain optical limiting. They measured a high ratio between the excited state absorption cross-section and the ground state one of about 20 times (five-times higher than our samples at 532 nm). Another work studying gallium, indium and thallium phthalocyanines, using 7-ns laser pulses at 532 nm, also observed good optical limiting performance [35]. Dini *et al.* [35] measured a ratio of about 13 times for indium and thallium phthalocyanines (three-times higher than our samples at 532 nm) and observed an optical limiting effect a little higher for Ga phthalocyanines. Shirk and co-workers [36] studied the excited state absorption of Pb phthalocyanines dissolved in chloroform using 8-ns laser pulses at 532 nm. They measured an excited state absorption cross-section spectrum with a magnitude approximately twice higher than the values reported here. Interestingly, the shape of the excited state absorption cross-section observed in [36] is very similar to the ones presented in this work. Gadolinium phthalocyanines dissolved in toluene have been studied by Vivas *et al.* [37] by using fs Z-scan to obtain the excited state absorption cross-section. At 530 nm, they measured a ratio between the ground and excited state absorption cross-section of about two (two-times smaller than the results presented here), although the highest ratio (~4) observed in [37] occurs around 500 nm. In the present work, however, it was possible to discriminate each individual pathway for an excited molecule, passing though excited singlet and triplet states. This allows one to predict that the optical limiting effect (at 532 nm) increases when ZnPcs are in the triplet state. The triplet state absorption cross-section are 10%, 12.5% and 15.5% for **B4**, **AB3** and **ABAB** respectively, higher than the singlet excited state ones.

## 3. Materials and Methods

### 3.1. General Information

Starting materials were purchased from Aldrich, Fluka and Alfa Aesar and used without further purification, unless otherwise stated. Residual water in zinc acetate salts Zn(OAc)_2_ was removed by heating at 120 °C under vacuum (10^−2^ mm Hg). 2,3,16,17-tetra(hexylthio)phthalocyanine (**ABAB-H2**) and 2,3,9,10,16,17 hexakis(hexylthio)phthalocyanine (**AB3-H2**) were prepared as previously described in [38], as well as (2,3,9,10,16,17,23,24-octakis(hexylthio)phthalocyaninato)Zinc (**B4)** in [39].

### 3.2. Synthesis: General Procedure for ***ABAB*** and ***AB3*** Metalation

A mixture of dried Zn(OAc)_2_ (0.1 mmol), DBU (0.7 mmol) and 0.1 mmol of **ABAB-H2** (or **AB3-H2**) in 1-hexanol (2 mL) was refluxed under argon atmosphere for 24 h. 1-hexanol was removed under reduced pressure, and the crude product was firstly purified by column chromatography over Al_2_O_3_ with a mixture of dichloromethane–methanol (30:1) and then by silica preparative thin-layer chromatography eluting with 20:1 dichloromethane–methanol (*v*/*v*), affording the pure products as a green solid. Yield: 30 mg for **ABAB** (30%), 25 mg for **AB3** (24%).

**ABAB**: C_56_H_64_N_8_S_4_Zn (1041.5). FT-IR (ν_max_ cm^−1^): 2958 (aliphatic C-H), 1664, 1594, 1437, 1388, 1260, 1061, 942. ^1^H-NMR (*d*_8_-THF, δ ppm): 8.99 (s, 4H), 8.78 (s, 4H), 8.07 (s, 4H), 3.50 (t, 8H), 2.11 (br s, 8H), 1.84 (br s, 8H), 1.59 (m, 16H), 1.07 (t, 12H). MS (MALDI-TOF, *m*/*z*): 1041.949 [M].

**AB3**: C_68_H_88_N_8_S_6_Zn (1273.4) FT-IR (ν_max_ cm^−1^): 2958(aliphatic C-H), 1661, 1597, 1432, 1386, 1260, 1064, 940. ^1^H-NMR (*d*_8_-THF, δ ppm): 9.09 (s, 2H), 8.82 (m, 6H), 8.16 (s, 2H), 3.49 (br s, 12H), 2.08 (br s, 12H), 1.88 (br s, 12H), 1.58 (br s, 24H), 1.06 (t, 18 H). MS (MALDI-TOF, *m*/*z*) 1274.437 [M + H]^+^.

### 3.3. Physical Measurements

The UV-VIS spectra were recorded in a 1-mm optical path length of a quartz cuvette on a UV-1800 SHIMADZU spectrophotometer within a range of 3000 nm in chloroform solution of $0.65\times 10^{17}$ molecules/cm^3^ (~1.08×10^−4^ mol/L) to sample **B4**, $0.64\times 10^{17}$ molecules/cm^3^ (~1.06×10^−4^ mol/L) to **AB3** sample and, finally, $0.49\times 10^{17}$ molecules/cm^3^ (~0.81×10^−4^ mol/L) to sample **ABAB**, respectively.

^1^H-NMR spectra were recorded on a Varian 500-MHz spectrometer (with the deuterated solvents as the lock and tetramethylsilane as the internal reference).

MALDI-MS spectra were obtained using 2,5-dihydroxybenzoic acid as the MALDI matrix, acquired in linear modes with an average of 50 shots on a Bruker Daltonics Microflex mass spectrometer equipped with a nitrogen UV-Laser operating at 337 nm.

Steady state fluorescence emission: Steady state fluorescence emission of all samples dissolved in chloroform was acquired by a HITACHI F7000 fluorimeter with a low concentration (on the order of 10^−6^ molar) in order to avoid re-absorption. Fluorescence quantum yield $\left(\phi_{f}\right)$ determination was achieved by the well-known Brouwer method, measuring the sample and a standard reference $\phi_{f}^{ref}$ under identical conditions, by exciting both of them at 630 nm. In our case, we used **B4** dissolved in THF as the reference sample ($\phi_{f}^{ref}=13\%$ as determined in [17]). In order to avoid re-absorption of the fluorescence by the sample, the optical density of these samples were chosen to be around 0.1 for a 1-cm optical path length of a square quartz cuvette (very low molecular concentration). Thus, the fluorescence spectrum of the reference and samples were measured by exciting both at the same wavelength (630 nm). Once fluorescence spectra were recorded, fluorescence quantum yields of the samples were obtained by using Equation (4):

(4) $$\phi_{f}=\phi_{f}^{ref}\;\times \;\frac{\int_{\lambda_{0}}^{\lambda_{f}}F\left(\lambda \right)\;d\lambda }{\int_{\lambda_{0}}^{\lambda_{f}}F_{ref}\left(\lambda \right)\;d\lambda }\;\times \;\frac{f_{ref}}{f}\;\times \;\frac{n^{2}}{n_{ref}^{2}}\;$$

in which F(λ) is the fluorescence spectrum as a function of the wavelength, which is integrated in a wavelength range that contemplates the full spectrum, f is the absorption factor, defined as $f=1-10^{-A\left(\lambda_{ex}\right)}$, such that $A\left(\lambda_{ex}\right)$ is the absorption of the sample at the wavelength excitation, n is the refractive index (1.442, chloroform) and $n_{f}$ is the reference refractive index (1.404, THF) [40].

#### Fluorescence Lifetimes (Time-Resolved Measurements)

In order to quantify the radiative lifetime of samples dissolved in chloroform, we measured fluorescence decay lifetimes $\left(\tau_{f}\right)$ employing as excitation 50-fs pulses at 680 nm and with a 1-kHz repetition rate, delivered by an optical parametric amplifier, which is pumped by a Ti:sapphire laser. The fluorescence produced by the sample was collected perpendicularly to the excitation laser beam (630 nm) through a 1-mm diameter large core optical fiber, positioned close to the fluorescent spot. The optical fiber directed the fluorescence signal to a fast silicon photodetector (D) with an ~700-ps rise time, which is fast enough to resolve the fluorescence decay. The photodetector electrical signal was acquired by a 1-GHz oscilloscope and recorded by a computer. This time-resolved fluorescence experimental setup is always calibrated by measuring Rhodamine 6G dissolved in ethanol. After acquiring the decay curves and the response time of the equipment, signal deconvolution is employed to obtain the fluorescence lifetime.

Z-scan experimental details: The nonlinear optics experiments were performed in a 2-mm optical path length of a quartz cuvette. For single and pulse train Z-scan measurements, the same concentrations as the one used in the linear absorption experiment were used ($0.65\times 10^{17}$ molecules/cm^3^ (~1.08×10^−4^ mol/L) to sample **B4**, $0.64\times 10^{17}$ molecules/cm^3^ (~1.06 × 10^−4^ mol/L) to **AB3** sample and finally $0.49\times 10^{17}$ molecules/cm^3^ (~0.81 × 10^−4^ mol/L) to **ABAB,** respectively). For the white light continuum Z-scan technique, a concentration 4-times lower was used.

Basically, the Z-scan technique consists of monitoring changes in the normalized transmittance (transmittance of the beam through the sample) while translating the sample through the focus of an intense laser beam (z-axis). The normalized transmittance is obtained by the ratio of the transmittance as a function of the z-position by the transmittance far from the focal plane (linear transmittance). This is a well-established technique, and more information about it can be found elsewhere [23,41,42].

The single pulse Z-scan technique at 532 nm uses a single pulse of a double frequency Q-switched and mode-locked Nd:YAG laser with 100 ps of pulse duration at a repetition rate of about 100 Hz. The spatial Gaussian profile is achieved by spatially filtering the beam with a 50-μm circular aperture (spatial filter). The lens used in the Z-scan setup has a focal distance of about 12 cm. The signal is collected using a silicon detector (PIN), and the electrical signal is averaged and amplified by a locking amplifier during 1 min. The average process is made for each individual point of the Z-scan. The data acquisition and Z-scan experimental control were carried out by homemade software based on LabVIEW. Especially, this setup allows measurements of the nonlinear optical effect (excited state absorption) at the picoseconds’ regime (only singlet states for the results described on this manuscript).

The pulse train Z-scan technique at 532 nm (PTZS) [24], uses a pulse profile (train) composed of approximately 25 pulses of double-frequency Q-switched and mode-locked Nd:YAG. Each individual pulse of the train presents a duration of 100 ps, and they are temporally separated by 13 ns. The measurements were made with a repetition rate of about 10 Hz to avoid thermal effects. This laser presents a temporal Gaussian profile, and a spatial Gaussian profile is achieved by spatially filtering the beam with a 50-μm circular aperture (spatial filter). The lens used in the Z-scan setup has a focal distance of about 12 cm. The signal is collected using a fast silicon detector (*ca.* 2 ns rise time) that allows one to monitor each individual pulse of the envelope. The electrical signal produced by the detector is averaged by a 1-GHz digital oscilloscope during 5 min. The averaged signal and Z-scan experimental control were carried out by homemade software based on LabVIEW. Especially, this setup allows mapping the nonlinear dynamics in the nanosecond time scale (used to determine the triplet state) due to the use of the fast detector. In this last one, while the sample is moved along the laser beam focal axis, as the standard technique, a train of pulses of a Q-switched/mode-locked laser is acquired. At the end of the measurement, a Z-scan signature is obtained for each individual pulse of the train. More information about this technique can be found in [24].

White light continuum Z-scan (WLCZS) [25,26,27,43]: Instead of a monochromatic excitation wavelength, this technique uses a broadband white light continuum pulse. The white light continuum is generated by focusing 150-fs pulses at 1110 nm in a 3-cm cell filled with distilled water. Typically, 1 μJ of WLC in the visible is generated by employing approximately 0.1 mJ at 1110 nm. The WLC beam is recollimated using a 10-cm focal distance lens, and a low pass filter is used to remove the pump pulse and part of the infrared portion of the WLC spectrum. A 7-cm focal distance lens is used on the Z-scan setup. The WLC spectrum employed in our experiment presents a bandwidth of approximately 350 nm, spanning from the visible (450 nm) to the NIR region (800 nm), with about 4 ps of positive chirp. The general procedure of the WLC Z-scan technique is the same as the conventional Z-scan. However, in the WLC Z-scan, a spectrometer (resolution of ~2 nm) is used instead of the photodetector generally employed in the standard technique, yielding Z-scan signatures for each wavelength.

## 4. Conclusions

Using different linear and nonlinear spectroscopic techniques, we were able to determine important spectroscopic parameters for a series of Zn(II) phthalocyanines dissolved in chloroform. The nonlinear optical techniques demonstrated that for the region between 450 and 620 nm, all samples present an excited state absorption cross-section greater than the ground state one. However, for wavelengths greater than 600 nm, the samples are more transparent than in the ground state. Such features indicate these samples as good candidates for optical limiting and either for saturable absorbers, dependent on the spectral region. A similar behavior was also obtained for the triplet state absorption at 532 nm; the ratio between the absorption cross-section at 532 nm indicates that when the molecules are in the triplet state, the efficiency of the optical limiting increases.

With respect to relaxation processes and quantum yield efficiency, all samples exhibited a moderate intersystem crossing process when dissolved in chloroform, with the highest triplet formation quantum yield of about 32% for **ABAB**. This indicates that the reduction of the hexyl chains increases the ISC time and decreases the fluorescence quantum yield. It was noticed that the main relaxation path occurs from the first singlet excited state to the ground singlet state by internal conversion, having a higher quantum yield efficiency of about 72% for **B4**.

These results confirm that Zn(II) phthalocyanines can be considered as suitable photosensitizers in the photodynamic therapy of cancer, because the triplet state formation mechanism is privileged.

## Figures and Tables

**Table 1:** Photophysical parameters of the studied ZnPc compounds dissolved in chloroform. Fluorescence lifetime ($\tau_{f}$), fluorescence quantum yield ($\phi_{f}$), radiative ($\tau_{r}$), non-radiative lifetime ($\tau_{\text{nr}}$), non-radiative ($\phi_{\text{nr}}$) quantum yield, intersystem crossing time ($\tau_{\text{isc}}$), triplet quantum yield ($\phi_{T}$) and radiative rate (k_r_).

Compound	$\tau_{f}$(ns)	$\phi_{f}$	$\tau_{r}$(ns)	$\tau_{nr}$(ns)	$\phi_{nr}$	$\tau_{isc}$(ns)	$\phi_{T}$	k_r_ (×10^7^ s^−1^)
**B4**	3.1 ± 0.2	0.096	32.3 ± 0.5	4.4 ± 0.2	0.717	16.5 ± 0.5	0.19	3.1
**AB3**	3.2 ± 0.1	0.181	17.7 ± 0.3	5.3 ± 0.1	0.608	15.1 ± 0.6	0.21	5.6
**ABAB**	3.0 ± 0.1	0.207	14.5 ± 0.2	6.5 ± 0.3	0.474	9.4 ± 0.5	0.32	6.9

## Supplementary Materials

Supplementary materials can be accessed at: http://www.mdpi.com/1420-3049/21/5/635/s1.

## Abbreviations

The following abbreviations are used in this manuscript:

ZnPcs	zinc (II) phthalocyanines
**B4**	2,3,9,10,16,17,23,24-octakis(hexylthio)phthalocyaninato)zinc
**AB3**	(2,3,9,10,16,17 hexakis(hexylthio)phthalocyaninato)zinc
**ABAB**	(2,3,16,17-tetrakis(hexylthio)phthalocyaninato)zinc
**ABAB-H2**	2,3,16,17-tetra(hexylthio)phthalocyanine
**AB3-H2**	2,3,9,10,16,17 hexakis(hexylthio)phthalocyanine
RSA	reverse saturation absorption
SA	saturable absorption
PTZS	pulse train Z-scan technique at 532 nm
WLCZS	white light continuum Z-scan
