# Supplementary material for: Mechanism of the Zn(II)Phthalocyanines’ Photochemical Reactions Depending on the Number of Substituents and Geometry
Source: Molecules. 2016 May 14;21(5):635. doi: 10.3390/molecules21050635 (PMC6274317; doi:10.3390/molecules21050635)
Supplement: Supplementary file 1 [file molecules-21-00635-s001.pdf]

Supplementary Materials: Mechanism of the Zn(II)Phthalocyanines' Photochemical Reactions Depending on the Number of Substituents and Geometry

Leandro Henrique Zucolotto Cocca, Mehmet Menaf Ayhan, Ayşe Gül Gürek, Vefa Ahsen, Yann Bretonnière, Jonathas de Paula Siqueira, Fernando Gotardo, Cleber Renato Mendonça, Catherine Hirel and Leonardo De Boni

AB3-H2

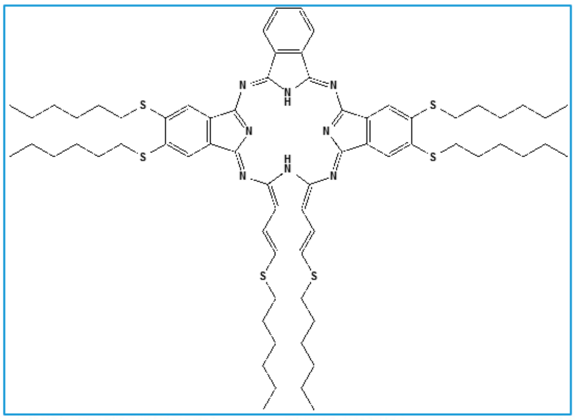

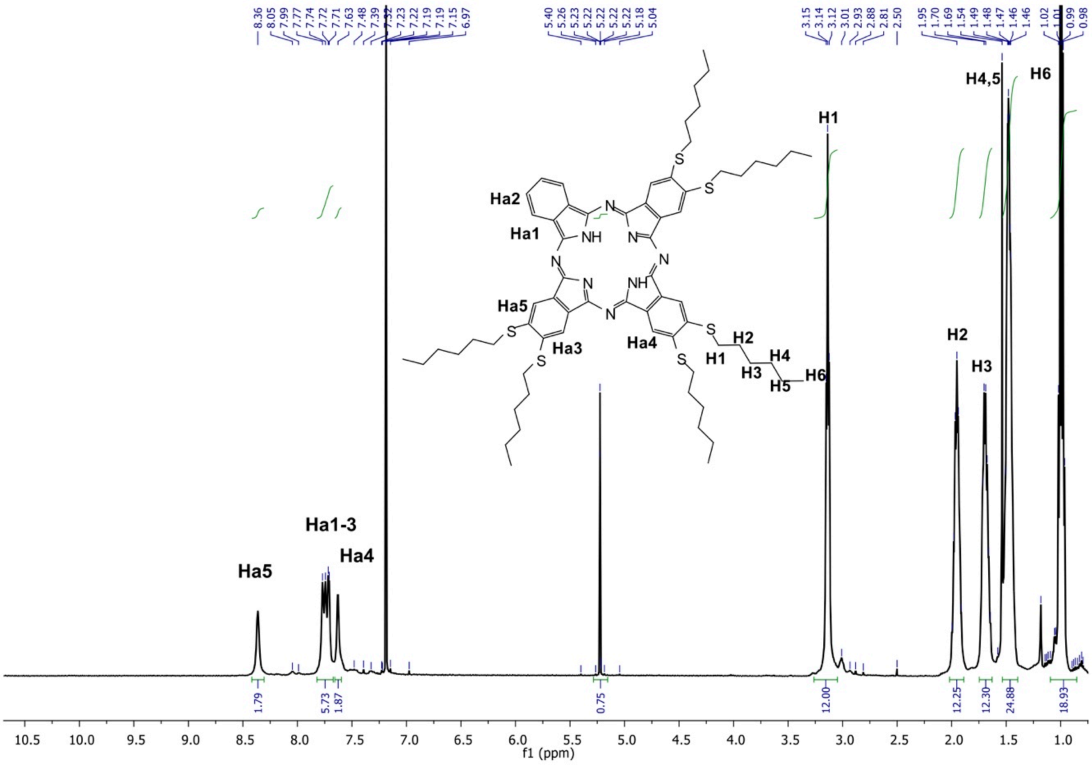

Figure S1. ^{1}H-NMR of **AB3-H2** in $CDCl_3$.

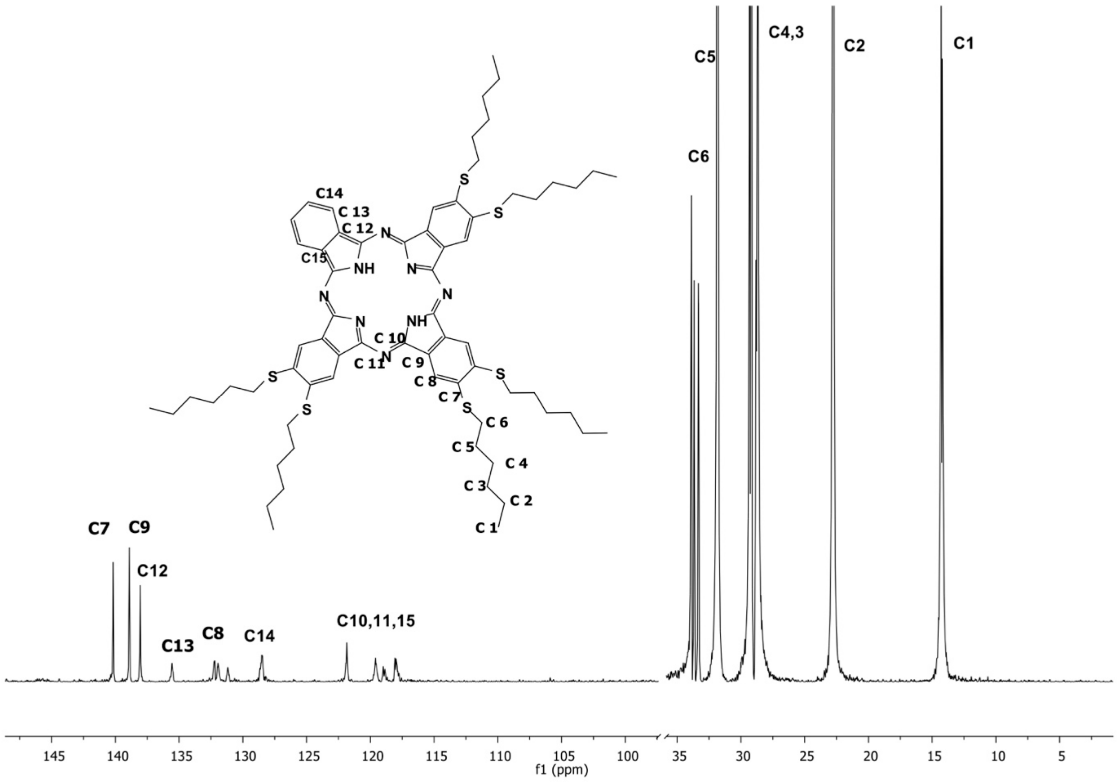

Figure S2. ^{13}C-NMR of **AB3-H2** in $CDCl_3$.

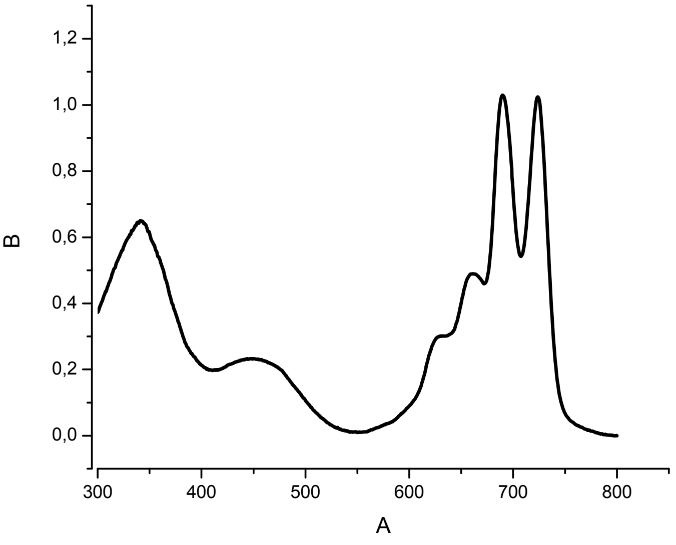

Figure S3. UV-VIS spectrum of **AB3-H2** in $CHCl_3$.

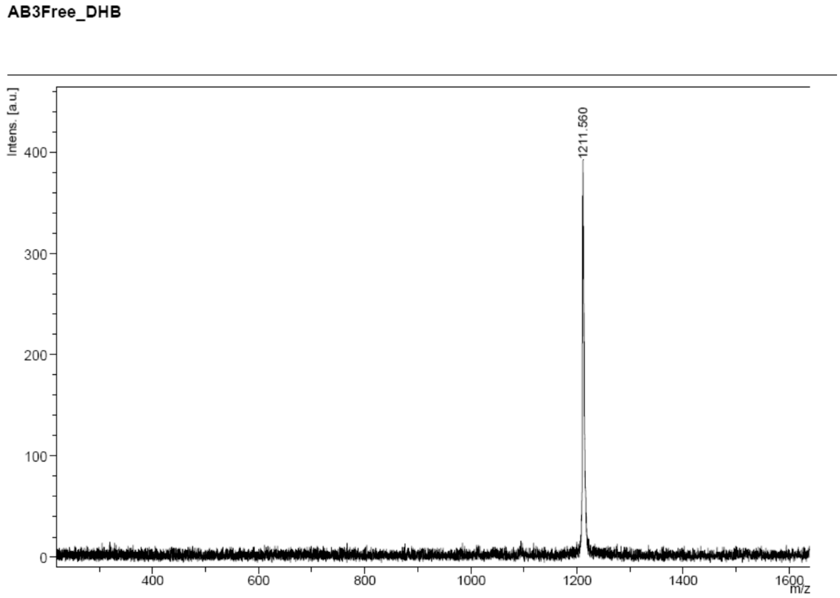

Figure S4. MALDI-MS with 2,5-dihydroxybenzoic acid as the matrix.

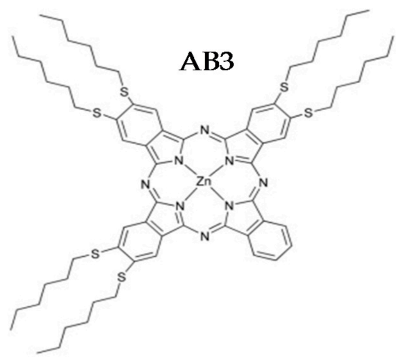

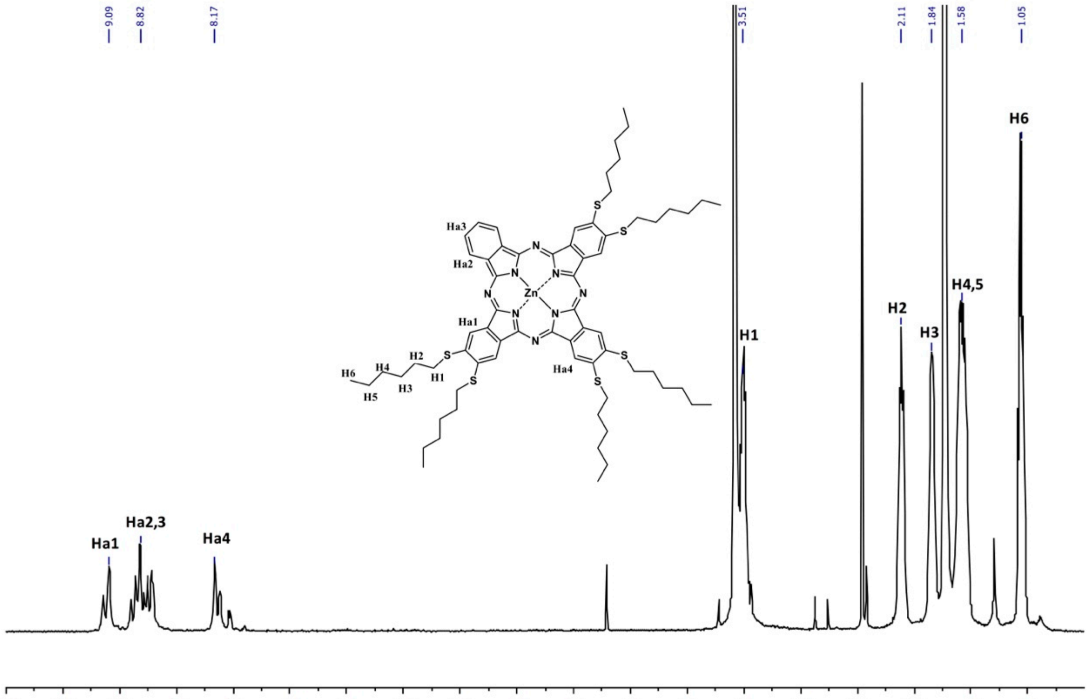

Figure S5. ^{1}H-NMR of **AB3** in d_8-THF.

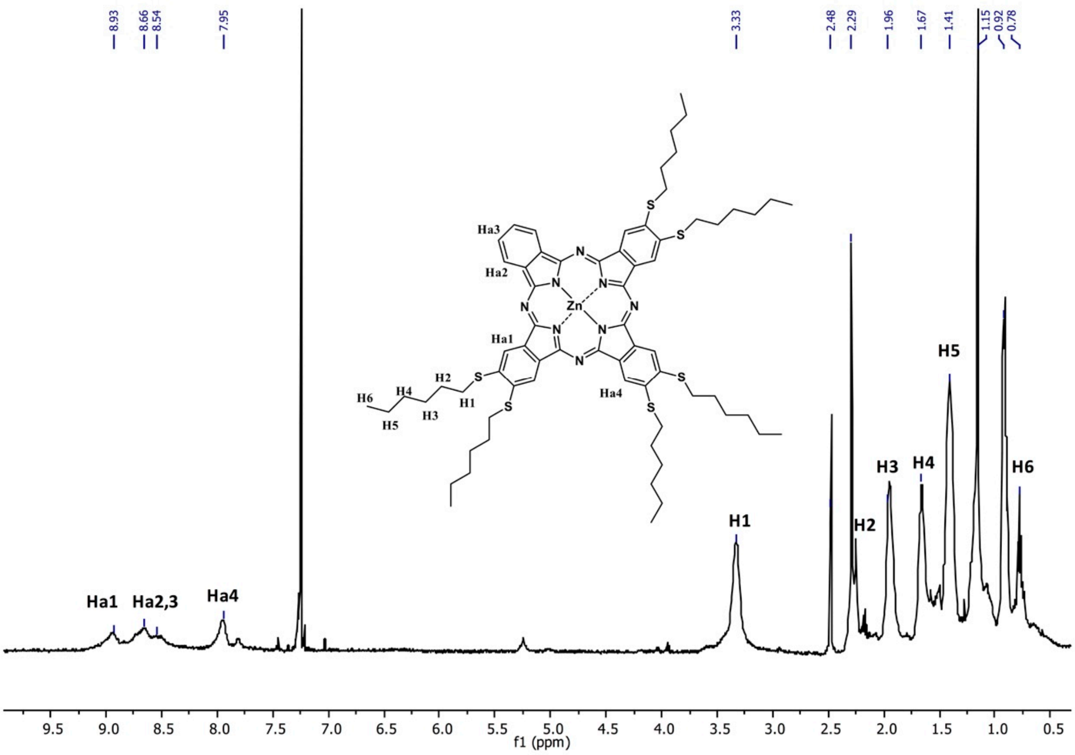

Figure S6. ^{1}H-NMR of **AB3** in $CDCl_3$ and DMSO.

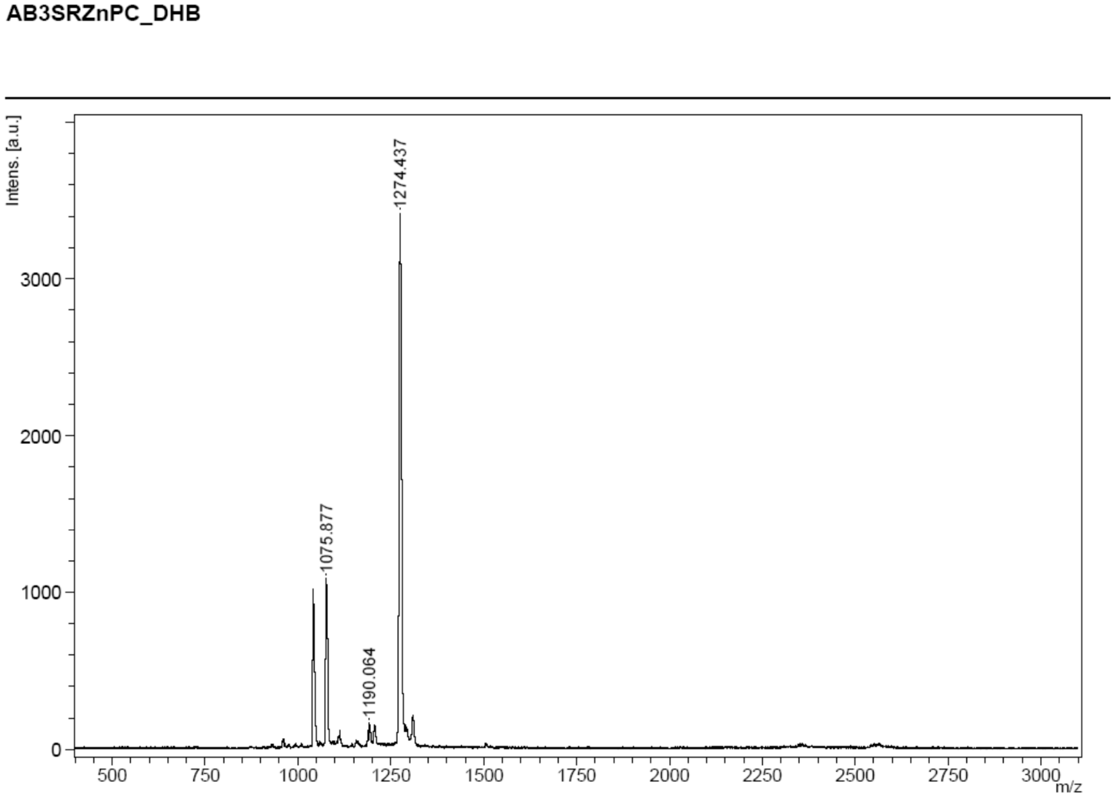

Figure S7. MALDI-MS with 2,5-dihydroxybenzoic acid as the matrix.

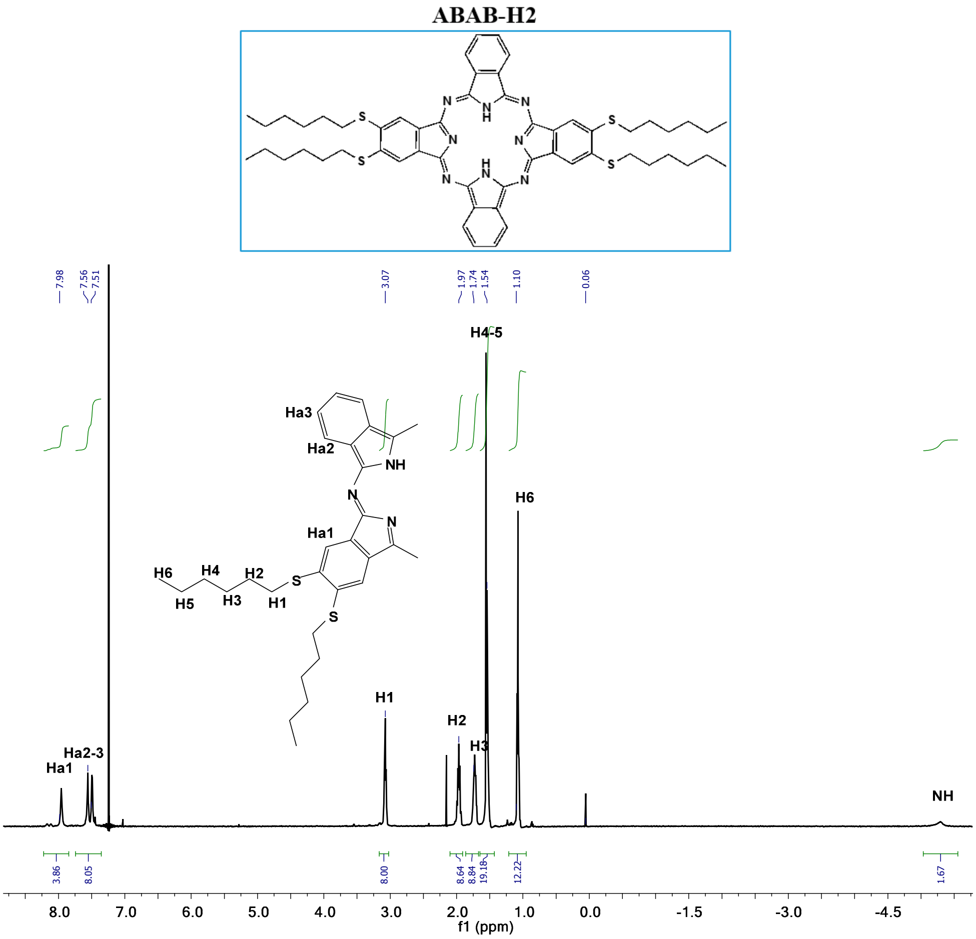

Figure S8. ^{1}H-NMR of **ABAB-H2** in $CDCl_3$.

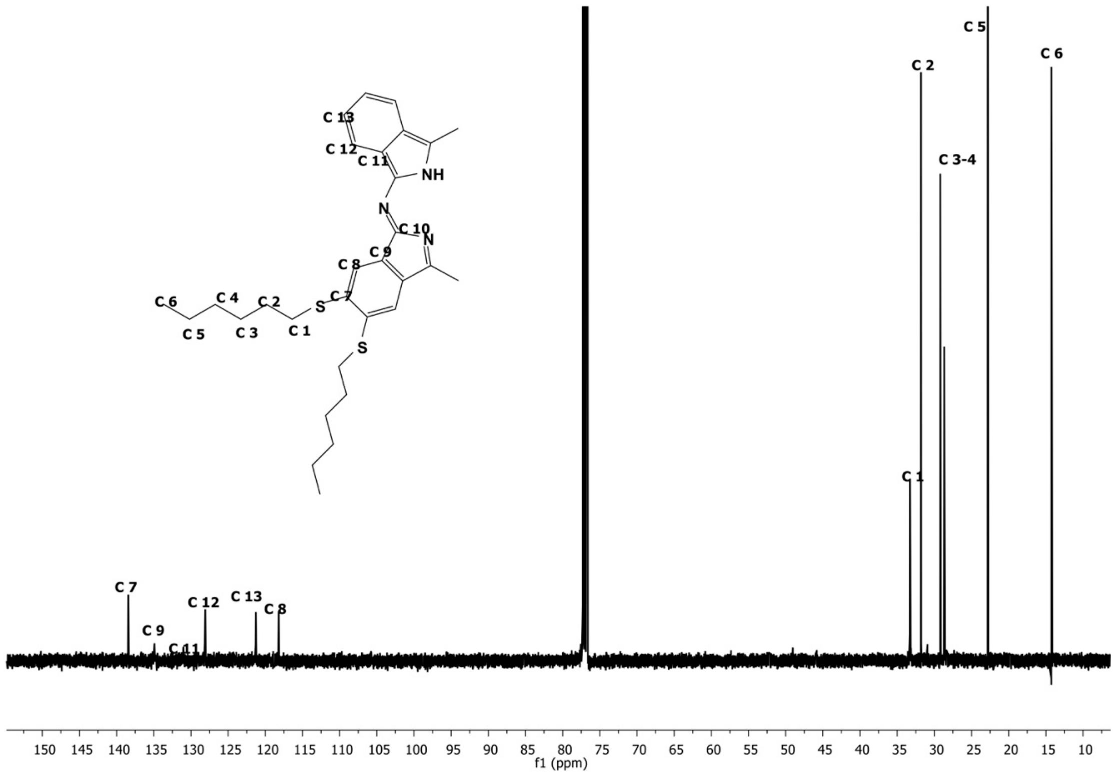

Figure S9. ^{13}C-NMR of ABAB-H2 in $CDCl_3$.

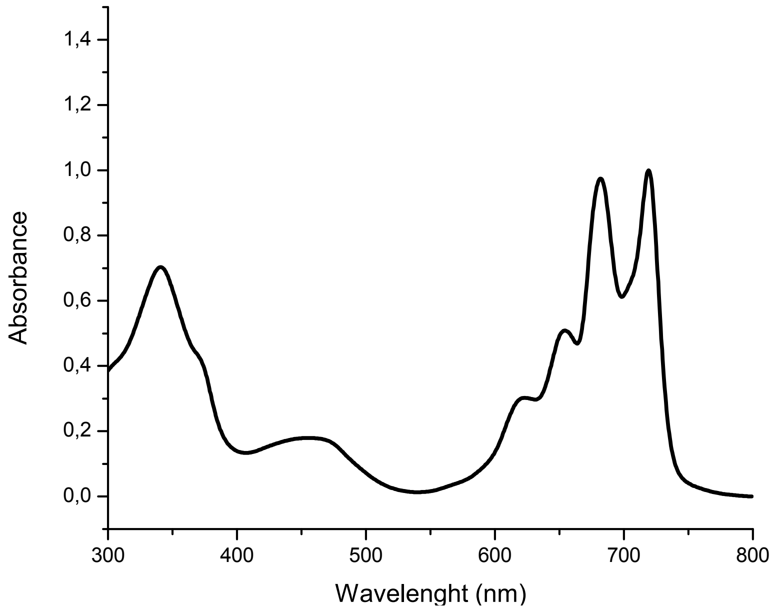

Figure S10. UV-VIS spectrum of ABAB-H2 in $CHCl_3$.

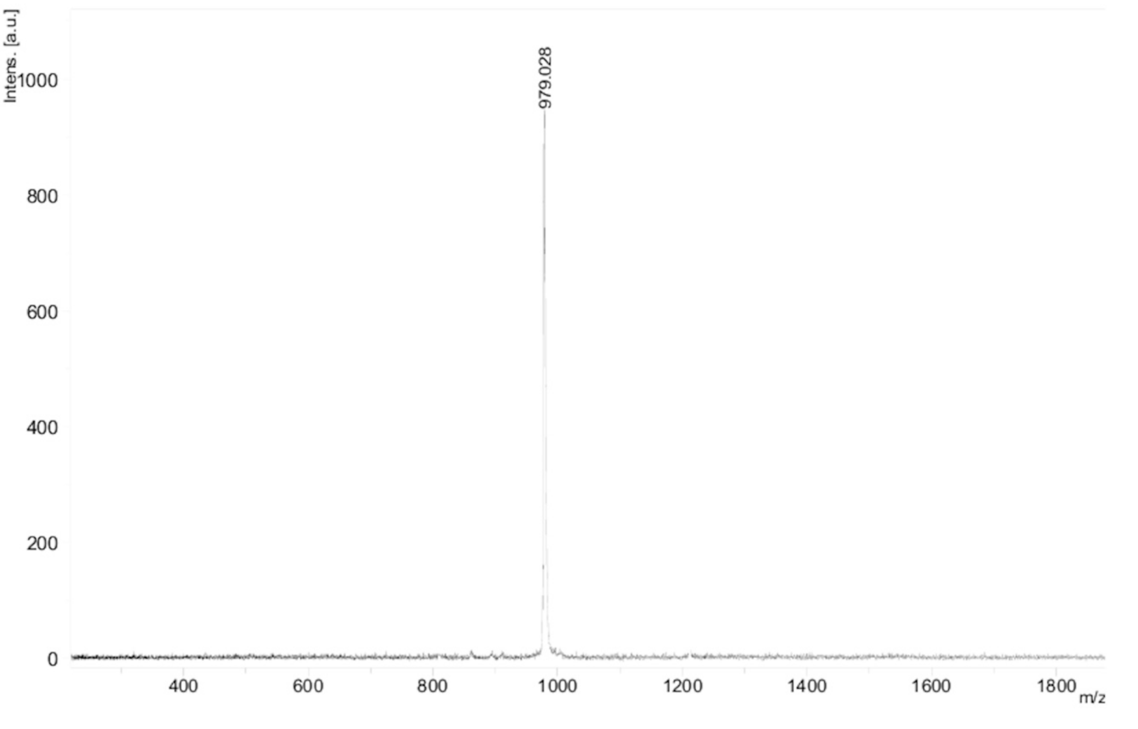

Figure S11. MALDI-MS with 2,5-dihydroxybenzoic acid as the matrix.

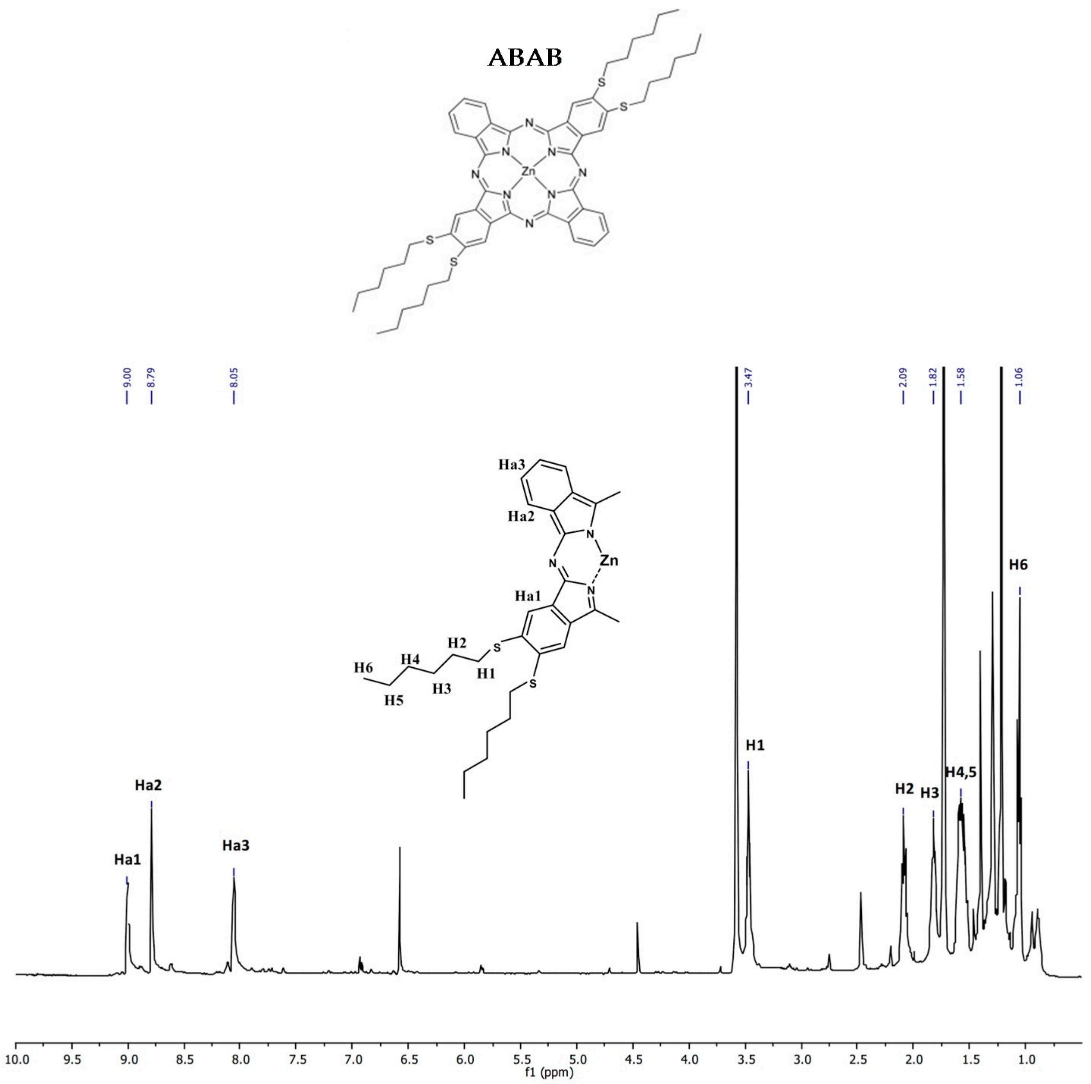

Figure S12. ^{1}H-NMR of **ABAB** in d_8-THF.

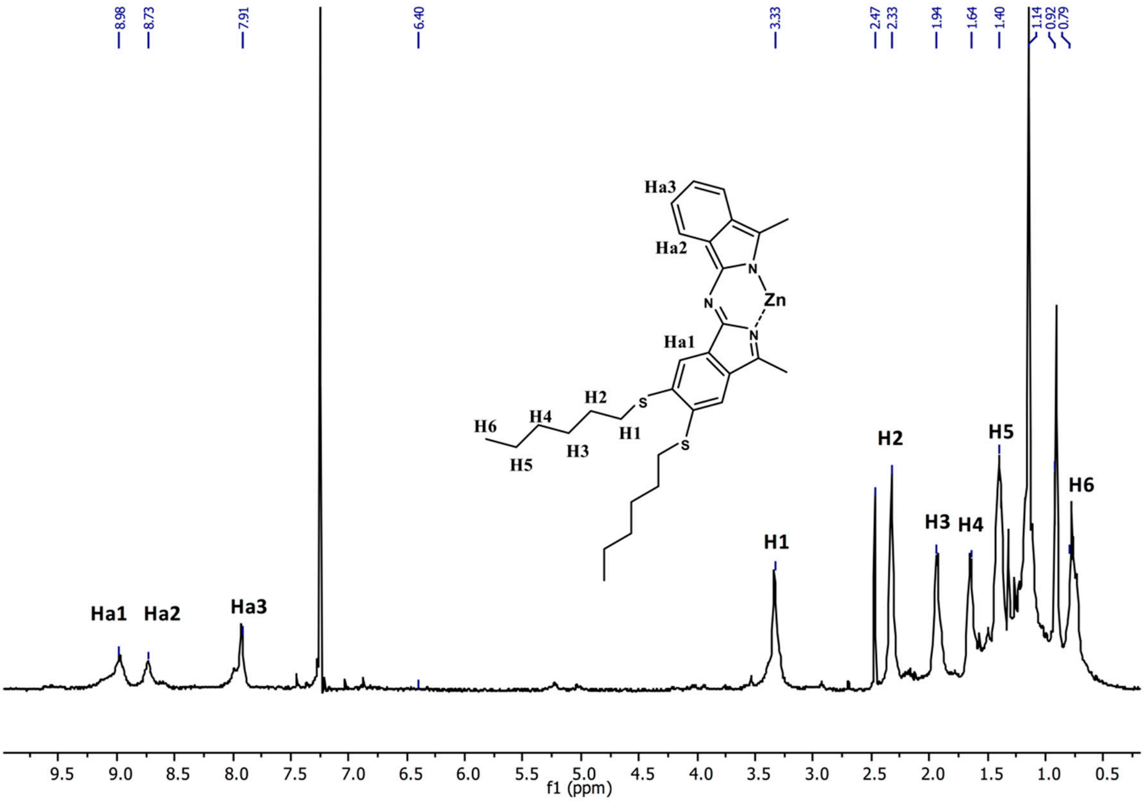

Figure S13. ^{1}H-NMR of **AB3** in $CDCl_3$ and DMSO.

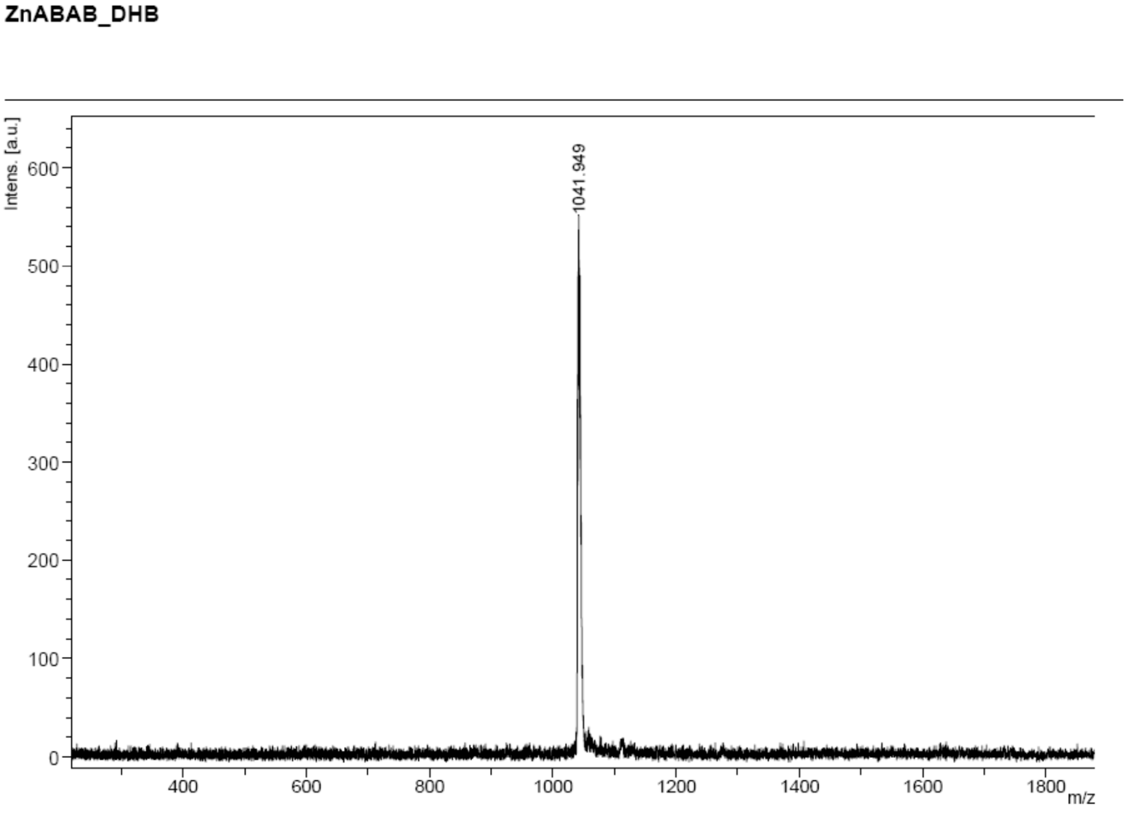

Figure S14. MALDI-MS with 2,5-dihydroxybenzoic acid as the matrix.
